# *DLEC1* is a functional 3p22.3 tumour suppressor silenced by promoter CpG methylation in colon and gastric cancers

**DOI:** 10.1038/sj.bjc.6604888

**Published:** 2009-01-20

**Authors:** J Ying, F F Poon, J Yu, H Geng, A H Y Wong, G-H Qiu, H K Goh, S Y Rha, L Tian, A T C Chan, J J Y Sung, Q Tao

**Affiliations:** 1 Cancer Epigenetics Laboratory, Department of Clinical Oncology, State Key Laboratory in Oncology in South China, Sir YK Pao Center for Cancer, Hong Kong Cancer Institute and Li Ka Shing Institute of Health Sciences, Chinese University of Hong Kong, Shatin, Hong Kong, China; 2 Department of Pathology, Cancer Institute and Cancer Hospital, Peking Union Medical College (PUMC), Chinese Academy of Medical Sciences, Beijing, China; 3 Department of Medicine and Therapeutics, Institute of Digestive Disease, Chinese University of Hong Kong, Shatin, Hong Kong, China; 4 Johns Hopkins Singapore, Singapore; 5 Yonsei Cancer Center, Yonsei University College of Medicine, Seoul, Korea; 6 Stanley Ho Center for Emerging Infectious Disease, Chinese University of Hong Kong, Shatin, Hong Kong, China; 7 Sidney Kimmel Comprehensive Cancer Center, Johns Hopkins School of Medicine, Baltimore, MD, USA

**Keywords:** tumour suppressor gene (TSG), DLEC1, CpG island, methylation, colon and gastric cancers

## Abstract

Promoter CpG methylation of tumour suppressor genes (TSGs) is an epigenetic biomarker for TSG identification and molecular diagnosis. We screened genome wide for novel methylated genes through methylation subtraction of a genetic demethylation model of colon cancer (double knockout of *DNMT1* and *DNMT3B* in HCT116) and identified *DLEC1* (Deleted in lung and oesophageal cancer 1), a major 3p22.3 TSG, as one of the methylated targets. We further found that *DLEC1* was downregulated or silenced in most colorectal and gastric cell lines due to promoter methylation, whereas broadly expressed in normal tissues including colon and stomach, and unmethylated in expressing cell lines and immortalised normal colon epithelial cells. *DLEC1* expression was reactivated through pharmacologic or genetic demethylation, indicating a DNMT1/DNMT3B-mediated methylation silencing. Aberrant methylation was further detected in primary colorectal (10 out of 34, 29%) and gastric tumours (30 out of 89, 34%), but seldom in paired normal colon (0 out of 17) and gastric (1 out of 20, 5%) samples. No correlation between *DLEC1* methylation and clinical parameters of gastric cancers was found. Ectopic expression of *DLEC1* in silenced HCT116 and MKN45 cells strongly inhibited their clonogenicity. Thus, *DLEC1* is a functional tumour suppressor, being frequently silenced by epigenetic mechanism in gastrointestinal tumours.

Tumorigenesis is a multistep process, with colorectal cancer (CRC) as the prototype model for multi-step genetic pathogenesis (Kinzler and Vogelstein, 1996). In this model, the key molecular event is the inactivation of multiple tumour suppressor genes (TSGs) due to genetic alterations. Also, it is now well established that alternative epigenetic silencing, such as methylation of promoter CpG islands (CGIs), leads to the inactivation of TSGs in virtually all tumour types and plays significant roles in tumour initiation and progression (Jones and Baylin, 2002). In CRC, epigenetic silencing of multiple TSGs has been reported frequently, including *MLH1, p16*^*INK4A*^*, MGMT, VHL, APC, RASSF1A, HIC1, CHFR, ADAMTS18* and *PCDH10* in various percentages of CRC tumours (Herman *et al*, 1998; Esteller *et al*, 2001; Kim *et al*, 2005; Ying *et al*, 2006; Jin *et al*, 2007 ). A growing list of TSGs with CGI methylation-mediated silencing has also been reported in gastric cancer (Leung *et al*, 2001). It is important to identify more new TSGs that are silenced by tumour-specific methylation in CRC and gastric cancers, which could serve as valuable biomarkers for molecular diagnosis and also provide clues to the molecular pathogenesis of these tumours.

In this study, we conducted a genome-wide search for genes with promoter methylation in CRC, by utilising CpG methylation-specific subtraction, in a CRC model of HCT116 cells deficient in *DNMT1* and *DNMT3B* (double knockout (DKO) cells). DNMT1 and DNMT3B are the two major DNA methyltransferases responsible for the maintenance and *de novo* CpG methylation, and the disruption of these two genes results in more than 95% loss of overall genomic methylation and CGI demethylation (Rhee *et al*, 2002). Virtually all known TSGs with methylation-mediated silencing in HCT116 became demethylated and reactivated in HCT116DKO, making it a good epigenetic model to identify novel candidate TSGs silenced in tumours (Rhee *et al*, 2002; Paz *et al*, 2003; Ying *et al*, 2005). Among the methylated target genes we identified, one is *DLEC1* (*Deleted in lung and oesophageal cancer 1*), located at 3p22.3 – a common tumour suppressor locus with frequent genetic abnormalities in multiple cancers (Imreh *et al*, 2003). The expression of *DLEC1* and its regulation in digestive tumours have yet to be evaluated. We found that *DLEC1* underwent promoter methylation-associated silencing in most CRC and gastric tumour cell lines and primary tumours, in a tumour-specific manner. Reintroduction of *DLEC1* into silenced tumour cells significantly suppressed tumour cell clonogenicity.

## Materials and methods

### Cell lines and primary tumours

Seven CRC (HCT116, HT29, LoVo, SW480, DLD1, LS180 and SW620) and 17 gastric cancer (Kato III, YCC1, YCC2, YCC3, YCC6, YCC7, YCC9, YCC10, YCC11, YCC16, SNU719, AGS, MKN28, NCI87, SNU1, SNU16 and MKN45) cell lines were used. Cell lines were routinely maintained in RPMI-1640 medium with 10% FBS. HCT116 cell line with genetic knockout of DNA methyltransferase genes (*DNMTs*): HCT116 DNMT1−/− (DNMT1KO), HCT116 DNMT3B−/− (DNMT3BKO) and HCT116 DNMT1−/− DNMT3B−/− (DKO) (gift from Dr Bert Vogelstein, Johns Hopkins) were grown with either 0.4 mg ml^−1^ genecitin or 0.05 mg ml^−1^ hygromycin or both (Rhee *et al*, 2002). DNA and total RNA were extracted from cell lines using TRI REAGENT (Molecular Research Center, Cincinnati, OH, USA). Genomic DNA of another five CRC cell lines (HCT15, RKO, SW48, Caco-2 and Colo205) and one immortalised normal colon epithelial cell line CCD-841 was also used. Genomic DNA samples from primary tumour tissues of 34 CRC and 89 gastric cancer patients were also used, with DNA samples of matched surgical marginal normal tissue samples from 17 CRC and 20 gastric cancer patients also available. Clinical information was available for all gastric cancer patients, including gender, differentiation, histological type according to Laurén and tumour, node and metastasis (TNM) stage. However, no survival data were available.

### Pharmacologic demethylation

Cell lines with silenced *DLEC1* were treated with 5 *μ*M of 5-aza-2′-deoxycytidine (Aza) (Sigma, St Louis, MO, USA) for 3 days as described earlier (Ying *et al*, 2005). After the treatment, cells were pelleted, with DNA and total RNA extracted.

### Modified methylation-sensitive representational difference analysis

To identify novel methylated TSGs, we employed a strategy of modified methylation-sensitive representational difference analysis (MS-RDA), using uracil-DNA glycosylase-based digestion during MS-RDA (Sugai *et al*, 1998; Kaneda *et al*, 2003), for DNA samples of the wild-type and DNA methyltransferases (DNMT1 and -3B) DKO of HCT116 (Rhee *et al*, 2002). The method was based on the principle that restriction enzymes (*Hpa*II, *Sac*II and *Nar*I) have different sensitivities towards sequences containing 5-methyl cytosine (CCGG, CCGCGG and GGCGCC). We further selected candidate genes with typical promoter CGI and also located at important chromosome loci commonly deleted in tumours and possibly harbouring putative TSGs for more detailed studies, such as *DLEC1*.

### Semi-quantitative reverse transcription PCR

Reverse transcription PCR (RT–PCR) was performed as described earlier (Tao *et al*, 2002; Ying *et al*, 2006), using *GAPDH* as a control. The primers for *DLEC1* are listed in Table 1. The PCR programme utilised an initial denaturation at 95°C for 10 min, followed by 33 cycles of reaction (94°C for 30 s, 55°C for 30 s and 72°C for 30 s), with a final extension at 72°C for 10 min.

### Bisulphite treatment and promoter methylation analysis

Bisulphite modification of DNA was carried out as described earlier using 2.4 M sodium metabisulphite (Tao *et al*, 2002). Methylation-specific PCR (MSP) and bisulphite genomic sequencing (BGS) were conducted according to our earlier reports (Tao *et al*, 1999; Ying *et al*, 2006). Methylation-specific PCR primers are listed in Table 1. Methylation-specific PCR was conducted at 95°C for 10 min, followed by 40 cycles of reaction (94°C, 30 s; 58°C for M, 55°C for U, 30 s; 72°C, 30 s), ended by 72°C for 5 min. Methylation-specific PCR primers were tested earlier for not amplifying any non-bisulphite-treated genomic DNA and thus specific. The MSP products of selected samples have been confirmed by direct sequencing. The top strand-specific BGS primers for bisulphite-converted single-stranded DNA of the *DLEC1* promoter are listed in Table 1. Amplified products were cloned into the pCR4-Topo vector (Invitrogen, Carlsbad, CA, USA), with six to eight colonies randomly chosen and sequenced.

### Cloning of the human *DLEC1* full-length open reading frame

Four pairs of primers were used to generate four *DLEC1* fragments based on the published *DLEC1* sequence (GenBank accession number AB020522): I, II, III and IV, which contain restriction enzyme sites of *Mfe*I, *Nde*I and *Apa*LI, respectively. The sequences of primers for these fragments are listed in Table 1. Reverse transcription was carried out using normal human testis RNA as a template (BD Biosciences, Palo Alto, CA, USA). Reverse transcription PCR products were cloned into pCR II-TOPO vector (Invitrogen) with the sequences and orientations confirmed from both ends. The four fragments were then ligated to form the full-length *DLEC1* cDNA, which was then cloned into the pcDNA3.1 vector, using the restriction sites *Bam*HI and *Mfe*I (vector and fragment I), *Mfe*I and *Nde*I (fragment II), *Nde*I and *Apa*LI (fragment III), and *ApaL* I and *Xho* I (fragment IV and vector), to generate the recombinant vector pcDNA3.1-DLEC1.

### Colony formation assay

Cells (1.5 × 10^5^ per well). were plated in a 12-well plate and transfected with either expression plasmid or the empty vector (0.8 *μ*g each), using FuGENE 6 (Roche Diagnostics, Mannheim, Germany). Forty-eight hours post-transfection, cells were collected and plated in a six-well plate, and selected for 2 weeks with G418 (0.4 mg ml^−1^). Surviving colonies (⩾50 cells per colony) were counted after staining with Gentian Violet. Total RNA from the transfected cells was extracted, treated with TURBO DNase (Ambion, Austin, TX, USA) and analysed by RT–PCR to confirm the ectopic expression of *DLEC1*. All the experiments were performed in triplicate wells for three times.

### Statistical analysis

Chi square test was used to analyse possible correlation between clinical parameters and *DLEC1* methylation status of tumour and non-tumour samples. For colony formation assay, experimental differences were tested for statistical significance using *t*-test. All analyses were performed using SAS for windows, version 9 software (SAS Institute Inc., Cary, NC, USA). A *P*-value of <0.05 was considered significant.

## Results

### Epigenetic identification of *DLEC1* as a methylated gene in CRC

**Figure 1:**
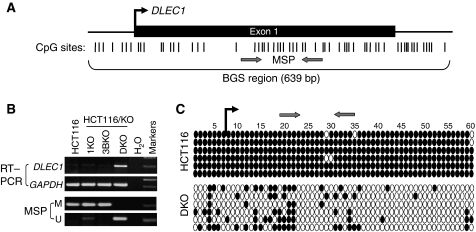
Identification of *DLEC1* as a methylated gene in CRC (HCT116) cells. (**A**) Schematic illustration of the *DLEC1* promoter and its CGI. Locations of the exon 1 (indicated with a long rectangle) and CpG sites (short vertical lines) in the CGI are shown. The transcription start site is indicated by a curved arrow. (**B**) Genetic demethylation reactivated *DLEC1* expression in DKO cells. U: unmethylated; M: methylated. (**C**) Detailed BGS analysis confirmed the MSP results. Methylation status of each individual promoter allele was shown as a row of CpG sites sequenced from each bacterium colony. Filled circle, methylated; open circle, unmethylated.

Using a modified MS-RDA to screen genome wide for methylated sequences in HCT116 and its demethylated DKO cells, we identified 22 hypermethylated DNA fragments/genes (Ying and Tao, manuscript in preparation). Among these identified sequences, one of particular interest is *DLEC1*, a candidate TSG previously identified in lung cancer (Daigo *et al*, 1999). Although no methylation was detected in this first report, the region spanning the putative promoter and exon 1 of *DLEC1* is a typical CGI (Gardiner-Garden and Frommer, 1987) (Figure 1A) that is susceptible to epigenetic silencing. We designed MSP and BGS primers to analyse its methylation status. Methylation-specific PCR analyses showed that *DLEC1* was completely methylated in HCT116 and became completely demethylated in DKO cells, but only marginally demethylated in DNMT1KO and not demethylated in DNMT3BKO cells. Correlated with its methylation status, *DLEC1* was silenced in HCT116, and only reactivated in DKO cells, but not in DNMT1KO or DNMT3BKO cells (Figure 1B). Detailed BGS analysis, revealing the methylation status of individual CpG site of the *DLEC1* promoter, showed that only few scattered CpG sites remained methylated in DKO cells whereas HCT116 was almost completely methylated (Figure 1C). These results thus demonstrate a close relationship between the silencing of *DLEC1* and its promoter methylation in HCT116 and DKO cells.

### Frequent methylation-associated silencing of *DLEC1* in CRC and gastric cell lines

**Figure 2:**
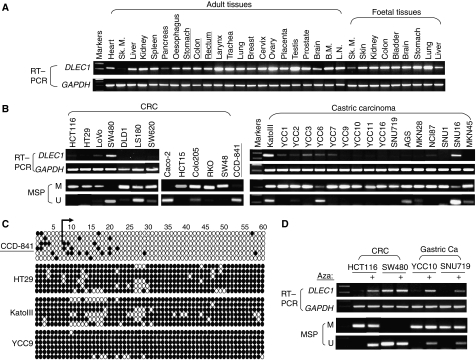
Frequent methylation-associated silencing of *DLEC1* in CRC and gastric cancer cell lines. (**A**) Expression profile of *DLEC1* in human normal adult and foetal tissues by semi-quantitative RT–PCR, with *GAPDH* as a control. Sk M, skeletal muscle; BM, bone marrow; LN, lymph node. (**B**) Methylation status and expression levels of *DLEC1* in a panel of CRC and gastric cancer cell lines. CCD-841 is an immortalised normal colon epithelial cell line. M, methylated; U, unmethylated. (**C**) Detailed BGS analysis confirmed the MSP results, as in Figure 1C. (**D**) Reactivation of *DLEC1* by Aza treatment (+), accompanied with demethylation of its promoter CGI.

To further examine the correlation of *DLEC1* methylation and silencing, we investigated additional human tissues and gastrointestinal cell lines. *DLEC1* was found to be readily expressed in all 22 normal adult and 9 foetal tissues including colon, rectum and stomach, with the highest level in testis and weak expression in skeletal muscle and pancreas (Figure 2A), in agreement with the earlier study that this gene is expressed in all tissues examined and abundantly in testis (Daigo *et al*, 1999). In contrast, *DLEC1* was silenced or downregulated in six of seven CRC and 15 of 17 gastric cancer cell lines (Figure 2B). By MSP, *DLEC1* methylation was detected in 83% (10 out of 12) of CRC and 100% (17 out of 17) of gastric cancer cell lines, with complete methylation detected in most cell lines, whereas no methylation was seen in the normal colon epithelial cell line CCD-841 (Figure 2B). Further BGS methylation analysis for one CRC, two gastric cell lines and CCD-841 confirmed the MSP results, with a high density of methylated CpG sites detected in all tumour cell lines, but not in CCD-841 (Figure 2C). Thus, the results revealed a strong correlation between *DLEC1* transcriptional silencing and its promoter methylation in virtually all CRC and gastric cancer cell lines examined, except for one CRC cell line LS180, which has both methylated and unmethylated promoter alleles but *DLEC1* expression is totally silenced, indicating that other mechanisms such as histone modification also could not be excluded.

### Restoration of *DLEC1* expression by pharmacologic demethylation

To determine whether methylation directly mediates the silencing of *DLEC1*, two CRC (HCT116 – methylated, SW480 – unmethylated) and two methylated gastric (YCC10 and SNU719) cancer cell lines were treated with Aza, a DNA methyltransferase inhibitor. After the treatment, *DLEC1* expression was restored in methylated cell lines along with an obvious increase of unmethylated promoter alleles (Figure 2D). In contrast, no significant change of *DLEC1* expression and methylation levels was observed in the unmethylated cell line SW480, indicating that Aza treatment did not cause indirect reactivation effect. Together with earlier results of *DLEC1* reactivation after genetic demethylation in DKO cells, these results showed that methylation of the *DLEC1* promoter directly leads to its silencing in CRC and gastric cancers.

### Frequent *DLEC1* methylation in primary CRC and gastric tumours

**Figure 3:**
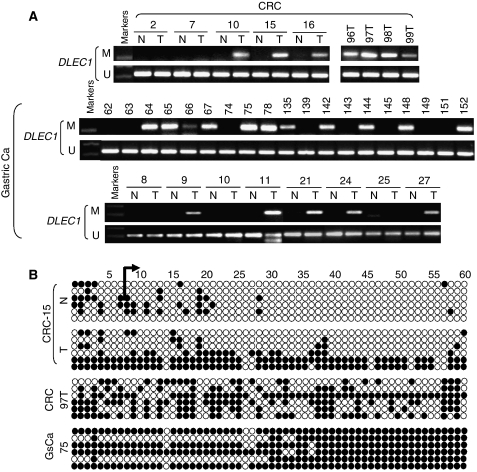
*DLEC1* methylation in primary CRC and gastric tumours. (**A**) Representative results of *DLEC1* methylation as detected by MSP in tumours (T), but not in the paired normal tissues (N). U: unmethylated; M: methylated. (**B**) High-resolution methylation mapping of individual CpG sites in the *DLEC1* CGI by BGS. GsCa: gastric carcinoma.

The methylation status of *DLEC1* was further examined in primary CRC and gastric tumour samples using the well-validated MSP analysis. Aberrant methylation was detected in 10 out of 34 (29%) of CRC and 30 out of 89 (34%) of gastric tumours, but seldom in paired normal gastric tissues (1 out of 20, 5%) nor any of the paired normal colon tissue samples (0 out of 17) (Figure 3A). Further BGS analysis revealed densely methylated promoter alleles in primary tumours, whereas only scattered methylated CpG sites in paired normal tissues (Figure 3B). Thus, promoter methylation of *DLEC1* is a frequent and tumour-specific epigenetic abnormality in CRC and gastric cancer.

Although the frequency of *DLEC1* methylation in gastric cancer was high (34%), no correlations between *DLEC1* methylation status and gender, tumour location, Lauren type, tumour differentiation and TNM stage were found (Table 2).

### Ectopic expression of *DLEC1* suppresses colorectal and gastric tumour cell clonogenicity

**Figure 4:**
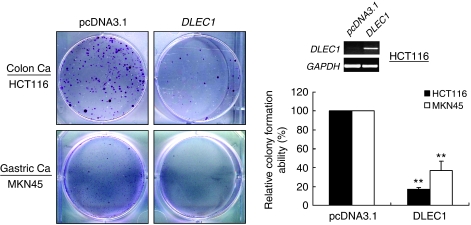
Ectopic *DLEC1* expression suppressed tumour cell clonogenicity of HCT116 and MKN45 cells. A representative inhibition of colony formation by *DLEC1* through monolayer culture assay is shown in the left panel. Ectopic *DLEC1* expression was determined by RT–PCR and quantitative analyses of colony numbers are shown in the right panel. Values are the mean±s.d. of three independent experiments. ^**^P<*0.01*.

The frequent silencing of *DLEC1* by methylation in colon and gastric cancer cell lines as well as primary tumours suggested that *DLEC1* is a potential tumour suppressor for these tumours. We thus investigated the tumour suppressor function of *DLEC1* by colony formation assay. The CRC cell line HCT116 and gastric carcinoma cell line MKN45 with silenced *DLEC1* were transfected with *DLEC1*-expressing vector pcDNA3.1-*DLEC1*. A strong reduction of colonies (i.e., down to 17 and 37% of the controls in HCT116 and MKN45 cells, respectively, *P*<0.01) was observed in cells transfected with pcDNA3.1-*DLEC1,* compared with the empty vector control (Figure 4). These results indicate that *DLEC1* indeed has growth inhibitory activities and could function as a tumour suppressor for colorectal and gastric cancer cells.

## Discussion

This is the first report to identify *DLEC1* as a methylated candidate TSG for CRC and gastric cancer. We demonstrated that *DLEC1* was absent in most colorectal and gastric cell lines due to promoter methylation, and methylated in a significant part of primary CRC and gastric tumours in a tumour-specific manner. In addition, the ectopic expression of *DLEC1* significantly suppressed colorectal and gastric carcinoma cell clonogenicity. Our results indicated that *DLEC1* is a functional TSG for CRC and gastric cancers, but is frequently inactivated by methylation-mediated silencing in these tumours.

Given the critical role of DNA methylation in the inactivation of TSGs as well as its potential application as tumour biomarkers for cancer diagnosis and prognosis assessment, various genome-wide techniques have been developed to screen for methylated genes in cancer cells. Among them, one is MS-RDA, which has been successfully used to identify methylated targets in multiple tumours (Kaneda *et al*, 2003; Ying *et al*, 2005). We used an improved MS-RDA to identify methylated targets in HCT116 comparing with DKO, in which virtually all known epigenetically silenced TSGs were reactivated with demethylation (Paz *et al*, 2003). The re-identification of multiple genes that had been shown earlier to be methylated in tumours represented a successful validation of this approach (Ying and Tao, manuscript in preparation). Considering promoter CGI and the chromosome location of identified genes, among the 22 targets identified, one regarded to be of particular interest is *DLEC1* (Daigo *et al*, 1999), which is located at the commonly deleted locus 3p22.3 and recently reported to be methylated in lung, ovarian and nasopharyngeal carcinomas (Kwong *et al*, 2006, 2007). Here, we provided solid evidence that *DLEC1* is also frequently methylated and acts as a functional TSG in CRC and gastric cancers.

Frequent deletion of 3p is one of the earliest molecular changes in tumours of the lung, nasopharynx, oesophagus, kidney, head and neck, breast, cervix and gastrointestinal tract (Hung *et al*, 1995; Kok *et al*, 1997; Wistuba *et al*, 2000; Hesson *et al*, 2007). Identification of TSGs in the gene-rich 3p22–21.3 region has been challenging, although several candidate TSGs within this region showed tumour suppressor functions, such as *RASSF1A* (Pfeifer *et al*, 2002), *SEMA3B* (Tomizawa *et al*, 2001), *BLU/ZMYND10* (Qiu *et al*, 2004), *FUS1* (Zabarovsky *et al*, 2002) and *HYA22 (RBSP3)* (Kashuba *et al*, 2004), with some of them (*RASSF1A* and *BLU*) frequently inactivated by promoter methylation-mediated silencing. *DLEC1,* also located at this region, contains 37 exons, spans ∼59 kb and encodes a 1755-amino-acid protein. *DLEC1* was first identified as a potential TSG involved in lung, oesophageal and renal cancers, but with no methylation detected (Daigo *et al*, 1999). Recently, *DLEC1* was reported to be frequently downregulated by methylation in ovarian and nasopharyngeal cancer (Kwong *et al*, 2006, 2007). Together with our results of *DLEC1* methylation in CRC and gastric cancers, *DLEC1* is likely to be inactivated in a wide range of tumours by promoter methylation and plays an important role in multiple tumorigenesis.

The function of *DLEC1* was further explored by examining the inhibitory effect of *DLEC1* expression on tumour cell growth. Introduction of *DLEC1* to silenced tumour cell lines HCT116 and MKN45 strongly suppressed their growth in colony formation assays. Similar tumour-suppressive properties were observed in oesophageal, renal and lung cancer cell lines (Daigo *et al*, 1999; Kwong *et al*, 2006). The proliferation and invasiveness of *DLEC1*-expressing cells were also greatly reduced with a dramatic reduction in tumorigenic potential in *in vivo* animal models (Kwong *et al*, 2007). These findings support that *DLEC1* is a functional TSG involved in multiple tumorigenesis; however, the mechanism underlying this role remains largely unknown. The predicted protein sequence of *DLEC1* has no significant homology to any known proteins or domains. Earlier report showed that 27 potential CK2 (formerly known as casein kinase II) phosphorylation sites are present in its predicted sequence (Daigo *et al*, 1999). Protein kinase CK2 is a pleiotropic, ubiquitous, constitutively active and second message-independent protein kinase and known to phosphorylate more than 100 substrates, many of which are involved in the control of cell division, signal transduction and many other cellular functions (Litchfield, 2003). CK2 is required at multiple transition in the cell cycle, including G0/G1, G1/S and G2/M (Litchfield, 2003), indicating that DLEC1 may be one of the increasing CK2 targets, modulated by CK2 and involved in cell cycle arrest.

Although *DLEC1* methylation has been shown to be associated with tumour stages in hepatocellular carcinoma (Qiu *et al*, 2008), we did not find any correlation between *DLEC1* methylation and clinical parameters in gastric tumours. A further larger scale study is needed to confirm this negative finding.

Tumour-specific promoter methylation can serve as a biomarker for tumour early diagnosis. The tumour-specific methylation of *DLEC1* in CRC and gastric cancers indicates that it could be used for such purposes in future. Our results revealed a higher frequency of methylation in cell lines (83–100%) but lower in primary tumours (29–34%), indicating that some cell lines may have acquired methylation during their establishment or maintenance process. Similar phenomenon has been reported for some other TSGs in other tumours as well (Smiraglia *et al*, 2001; Paz *et al*, 2003; Ying *et al*, 2005).

In summary, we found that *DLEC1* is frequently silenced by promoter methylation in colorectal and gastric cancers in a tumour-specific manner. We also showed that the methylation-mediated silencing of *DLEC1* could be reversed by genetic or pharmacologic demethylation, and restoration of *DLEC1* suppressed tumour cell clonogenicity, providing significant evidence that *DLEC1* functions as a tumour suppressor in these tumours. It would thus be worthy further exploring the possible use of *DLEC1* methylation as an epigenetic biomarker for molecular diagnosis.

## Figures and Tables

**Table 1:** List of primers used in this study

**PCR**	**Primer**	**Sequence**	**Location**	**Product size**	**PCR cycles**	**Annealing temperature (°C)**
*RT–PCR*	DLEC1A	ttcctccctcgcctactc	Exon 1	309 bp	33	55
	DLEC1B	aaactcatccagccgctg	Exon 2			
*cDNA cloning*	Fragment I	gccgccaccatggagaccagggc	Exon 1	∼1.1 kb	35	53
		gtgaaaaacccaattggtgg	Exon 6			
	Fragment II	agtgtttctagctaagccac	Exon 6	∼1.2 kb	35	53
		gagggcatatggctctaag	Exon 14			
	Fragment III	cttagagccatatgccctc	Exon 14	∼1.4 kb	35	53
		gccatgtgcactgggatg	Exon 25			
	Fragment IV	catcccagtgcacatggc	Exon 25	∼1.6 Kb	35	53
		gctcgagcggagcctcaggg	Exon 36			
*MSP*	DLEC1m1	gtttcgtagttcggtttcgtc	Exon 1	107 bp	40	58
	DLEC1m2	cgaaatatcttaaatacgcaacg	Exon 1			
	DLEC1u1	tagttttgtagtttggttttgtt	Exon 1	110 bp	40	55
	DLEC1u2	acaaaatatcttaaatacacaaca	Exon 1			
*BGS*	DLEC1BGS1	cgaagatataaatgtttataatgatt	Promoter	597 bp	40	55
	DLEC1BGS4	caactacaaccccaaatcctaa	Intron 1			

BGS=bisulphite genomic sequencing; MSP=methylation-specific PCR.

**Table 2:** Clinicopathologic features of *DLEC1* methylation in gastric cancer

**Variable**	**Methylated (*n*=30)**	**%**	**Non- methylated (*n*=59)**	**%**	***P*-value**
*Gender*					
Male	20	40.8	29	59.2	0.843
Female	10	38.5	16	61.5	
					
*Location*					
Corpus	23	35.9	41	64.1	0.476
Antrum	7	28.0	18	72.0	
					
*Lauren*					
Diffuse	4	18.2	18	81.8	0.131
Intestinal	19	35.8	34	64.2	
					
*Differentiation*					
Poor (or no differentiation)	19	38.0	31	62.0	0.422
Well or moderate	11	29.7	26	70.3	
					
*TNM (tumour, node, metastasis) stage*					
i and ii	15	38.5	24	61.5	0.440
iii and iv	15	30.6	34	69.4	
